# What’s in a name? A naming convention for geomorphic river types using the River Styles Framework

**DOI:** 10.1371/journal.pone.0201909

**Published:** 2018-09-19

**Authors:** Kirstie A. Fryirs, Gary J. Brierley

**Affiliations:** 1 Department of Environmental Sciences, Macquarie University, Sydney, Australia; 2 School of Environment, The University of Auckland, Auckland, New Zealand; Centro de Investigacion Cientifica y de Educacion Superior de Ensenada Division de Fisica Aplicada, MEXICO

## Abstract

Meaningful iteration between place-based knowledge of rivers and generalised, theoretically-framed understandings is a significant challenge in river science and management. How can we communicate knowledge of the inherent complexity of river systems in light of managerial quests for simple, easy-to-apply frameworks that can be used by a wide range of practitioners, such that we can meaningfully transfer experiences in river science and management from one situation to another? Identification, definition, classification and naming are vital parts of this process. In a sense, a name is like a ‘brand’, for which a consistency of product is expected. The River Styles Framework is a flexible, open-ended approach to river science and management. The Framework applies a set of hierarchical principles to differentiate reaches, interpret their process-based behaviour and examine interactions between patterns of reaches at the catchment scale. Here we outline an evolution and tightening of the Framework to better communicate how to identify and name types of river at the reach scale. Like the River Styles Framework itself, the naming convention applies hierarchical procedures, starting at the valley setting scale, and incorporating analyses of river planform, channel and floodplain landforms (geomorphic units) and bed material texture. Using a series of examples from around the world, we show how this naming convention can be applied to name river reaches and can be adapted to particular purposes in a consistent, readily communicable manner. We outline various challenges that are faced in managing the use of such a naming convention.

## Introduction

In recent decades there has been greater recognition of the range and importance of geomorphic river diversity, extending beyond simple differentiation of self-adjusting alluvial rivers (e.g. [1, 2]) and bedrock rivers (e.g. [3]) to include a range of partly-confined variants (e.g. [4, 5]). In light of this variability, how effectively do we communicate our understandings of rivers across the spectrum of river diversity, especially considering human-induced constraints upon river character and behaviour? A carefully framed naming convention is required to transfer understanding from one location or situation to another, aiding comparison of like-with-like. This paper proposes a clear, systematic and consistent approach to the naming of river types.

The lexicon of fluvial geomorphology is laden with terminology that can often confuse even the most engaged and knowledgeable practitioner [6]. Changing uses of names and terms may result in loss of their original meaning [7, 8]. If a term is ***misinterpreted***, it may be used in ways that were not intended. In other instances, a term is ***non-transferrable*** and a new term is developed to describe and explain phenomena in different contexts. Alternatively, ***replacement*** may occur, whereby a new term is assigned to describe or explain the same thing. In many instances, a plethora of terms may describe the same phenomenon. This equifinality of terms limits effective communication in river science and management.

Geomorphologists are challenged to develop a language that helps us to visualise and communicate understandings of particular landforms and/or landscapes [9, 10, 8]. The description and measurement of river planform [11], or the classification of bars [12], or the differentiation of floodplain types [13] requires a lexicon that provides an immediate snapshot image or descriptor of what a landform or landscape looks like. At the same time, rapid development of process-based interpretations of landscape adjustment and change results in a lexicon that directly (often implicitly) links form and process [7]. Often new names are assigned to landforms, landscapes and processes either because they had not previously been described, no naming convention existed, or the practitioner was not aware of what had gone before. More commonly, many names have local meaning that are not necessarily part of the scientific lexicon, yet they may provide locally nuanced understandings of how certain landforms were formed and how they function (e.g. terms such as billabong and cutoff, floodrunner and floodchannel). Indeed, the various glossaries of landmarks presented by Macfarlane (2015) [14] provide an expressive sense of meaning about the character and behaviour of landscape features. Among many examples, it is interesting to contemplate terms such as *aghlish*–a Manx term for a crook or sharp curve in a river (literally, ‘armpit’), *bathshruth*—an Irish term for a calm, smoothly flowing stream, *berw*–a Welsh term for boiling (foaming) water, *burraghlas*–a Gaelic term for a torrent of brutal rage, *caol*–and Irish term for a stream flowing through a marsh, and *pow*–a Cumbrian term for a sluggish, slow-moving stream with a muddy bottom.

It is one thing to encourage scientific exploration through open-ended approaches to enquiry, and quite another to reflect upon the equivalence of meaning that results [15, 8]. In the same vein as the question: “How long is the coastline of Britain?” [16], it is far from a pedantic issue to consider: “What is a mountain (or particular landform type)?” [17, 18]. Such questions draw attention to how the boundary of a feature is defined and how the scale of analysis is determined. Effective naming of particular landforms or river reaches has implications for the meaning that is conveyed, and in some instances the process-based interpretations that result and the management actions that ensue [19]. While implementation of management strategies based upon particular ‘types’ of river reach may facilitate straightforward applications of management procedures, they may also promote overly simplified applications that may produce negative consequences [20, 21]. The designation of river ‘types’ embeds particular sets of values, often with significant implications for the on-ground work that is done [22]. Lewin (2016) [7] asks geomorphologists to consider carefully what is named (as this is what gets discussed) and to consider whether the lexicon is sufficiently clear and reliable to convey true meaning, while also recognising that the lexicon is never complete.

Here we contend that it is timely to re-examine the development and use of naming conventions in river science and management. On the one hand, we feel it is important to ensure clarity of communication and meaning. On the other, we seek to promote consistency in terminology across the spectrum of riverscapes, supporting effective use of terminology among different audiences. This does not mean starting again (i.e. forgetting past research and using a clean slate). Although fluvial geomorphologists have developed a sophisticated understanding of controls upon river diversity, with significant agreement amongst classification frameworks as to the primary ‘types’ of river [23], there is little agreement in the terminology used to name these types. Although different frameworks may have emerged in different parts of the world, we feel it is important that practitioners have a standardised scheme for naming the same type of river in the same way. Having said this, such schemes should not be used in an unduly prescriptive manner, wherein particular situations are ‘forced’ into a particular category [24].

Meaningful differentiation can be made between classification procedures which apply prescriptive conventions (such as dichotomous keys) to classify, identify and name river types, relative to characterisation procedures that relate a given field situation to a generalised model, and test the degree of fit relative to other models [25]. While rule based classification procedures work inwards from the boundaries of a class, an archetype framework endeavours to define a central tendency of a class, working outwards for any given situation to assess how close that instance fits in relation to a central tendency (see [25, 26]). In a rivers context, classes produce bounded categories of reaches, whereas archetypes define core traits of reaches. While some sections along a reach are ‘perfect’ examples of a particular river type, other sections may not be quite so ‘perfect’ [26, 27]. A given reach may be a perfect example of a particular river type at one flow stage, but may not be quite so perfect as the river adjusts during a flood event.

In light of these considerations, process-based naming conventions must recognise and incorporate understandings of spatial and temporal variability. Indeed, just as genetics research draws into question classes of species, family and genera (e.g. recent work on acacia; [28]), an adaptive (learning) approach to naming of geomorphic river types based on their form and process is required. The convention designed here, and the resultant names, communicate the core, diagnostic attributes of the river as part of a characterisation framework. The benefits afforded by flexible approaches to river characterisation relative to classification are substantive. However, in both instances, it is vital to apply consistent procedures in identifying a reach and defining a name for that type of river.

In this paper we reflect upon various issues that have arisen in the characterisation and naming of river types through experiences gained in the development and uptake of the River Styles Framework [29]. In this paper, we align the procedural attributes of the River Styles Framework with a convention for identifying and naming river types. River Styles are reaches of river with a characteristic character and behaviour (form and function) [29]. Like the framework itself, we propose an open-ended but structured procedure to name river types across the spectrum of river diversity, wherein new variants can be added, while maintaining consistency in the naming of river types already identified in various parts of the world. We focus on ‘articulation’ to understand the building blocks that make up river type names in efforts to develop a reliable reference system that can be used as a communication tool among a range of audiences and practitioners (cf., [6, 30, 31]). Prospectively, the use of intuitive procedures can help to break down communication barriers, reducing the ‘turbulence and trainwrecks’ that often characterise management practice [32, 33] by establishing a convention and name that aids ‘mobility of meaning’ [7, 34, 35]. As semi- and fully-automated approaches to analysis and identification of landforms, river reaches, valleys and other features come to the fore (e.g. [36, 37, 38]), we think it is important to outline foundational principles and procedures that can support (or underpin) such applications and the assignation of names to outputs that are produced.

### Results: A naming convention for river reaches and river types

The identification, naming and presentation of River Styles is a four step process, each of which is defined in further detail in the following sections:

1. **The problem**–correctly differentiate and interpret river reaches at the outset.
2. **The procedure**–the framework and measures used to distinguish and differentiate river types, allowing for characterisation of new types if required.
3. **The convention**–the sequence of steps used to name different river types in a consistent manner, irrespective of landscape setting.
4. **The product**–a standard approach for assigning full (verbose) and abbreviated names for different river types that can be applied across the spectrum of river diversity.

### The problem: Correctly differentiate river reaches at the outset

**Fig 1:**
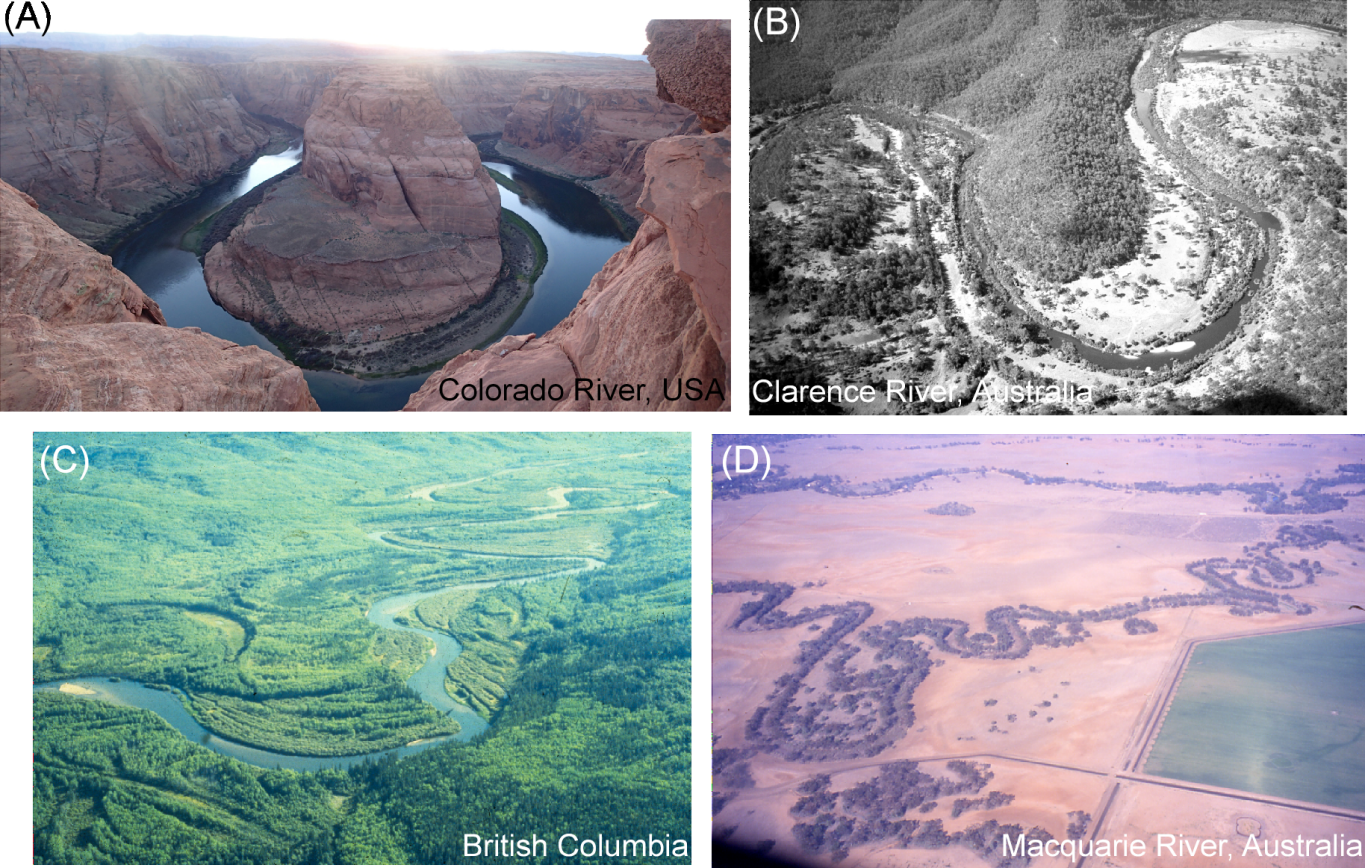
Are these all meandering river types? See text for explanation. Images sourced from the personal photograph archives of the authors. Reproduced with their permission. (A, D) Kirstie Fryirs, (B, C) Gary Brierley.

There is no magic number of river types [39]. Although various end members can be identified along the spectrum of variability from bedrock to fully alluvial rivers (including discontinuous watercourses), some conventional names create challenges to clear interpretation. In part this reflects variability in what particular rivers look like, but it is even more challenging in terms of their behaviour (i.e. the types, patterns and rates of geomorphic adjustment that define the capacity for adjustment and the range of variability of a given reach; see [29, 40]). Therefore it is critical that users spend time considering the range of possibilities from the outset. For example, consider channels with a ‘meandering’ outline. **Fig 1A** is a confined river, in which the ‘meandering’ channel outline is imposed by bedrock. This sinuous canyon has limited capacity for geomorphic adjustment. **Fig 1B** has a low sinuosity channel within a sinuous bedrock valley. River morphology is imposed by bedrock valley margins and the channel has limited capacity for geomorphic adjustment. Discontinuous pockets of floodplain occur on the insides of bends. This is a partly confined river [4, 5]. **Fig 1C** is an active meandering river. It has point bars on the inside of bends, along with ridges and swales, cutoffs and ox-bow lakes (billabongs) on its floodplain [41]. This mixed load river has significant capacity to adjust. **Fig 1D** is a passive meandering river. The channel is sinuous but there are no (or very limited) instream geomorphic units and the floodplain is flat, comprised solely of vertical accretion deposits. This suspended load river adjusts very slowly.

**Fig 2:**
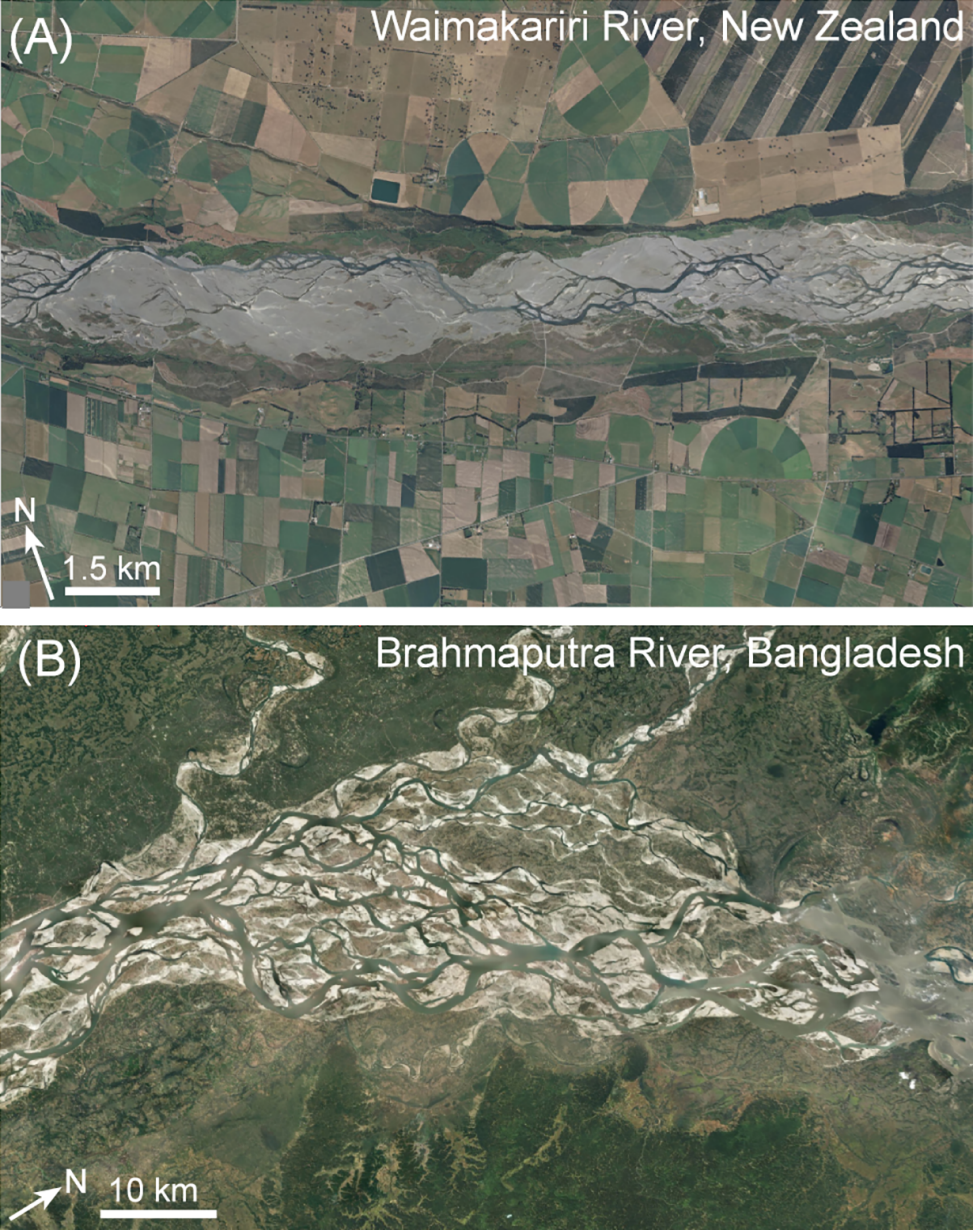
Are these all braided river types? See text for explanation. Basemaps produced with ArcGIS® software by Esri. Sources: Esri, DigitalGlobe, GeoEye, Earthstar Geographics, CNES/Airbus DS, USDA, USGS, AeroGRID, IGN, and the GIS User Community.

The same sorts of issues arise when considering ‘braided’ rivers (see [42, 43]). **Fig 2A** shows a braided river that has incised into alluvial materials, such that the contemporary channel sits within a ‘trough’ with adjacent margins defined by terraces. Given the pockets of contemporary floodplain, this is a partly confined river (see [44]). This contrasts significantly with braided rivers that occur on alluvial fans or in relatively steep but sufficiently wide alluvial valleys (**Fig 2B**). These laterally unconfined rivers have significant capacity to adjust, as channels are prone to avulsion.

**Fig 3:**
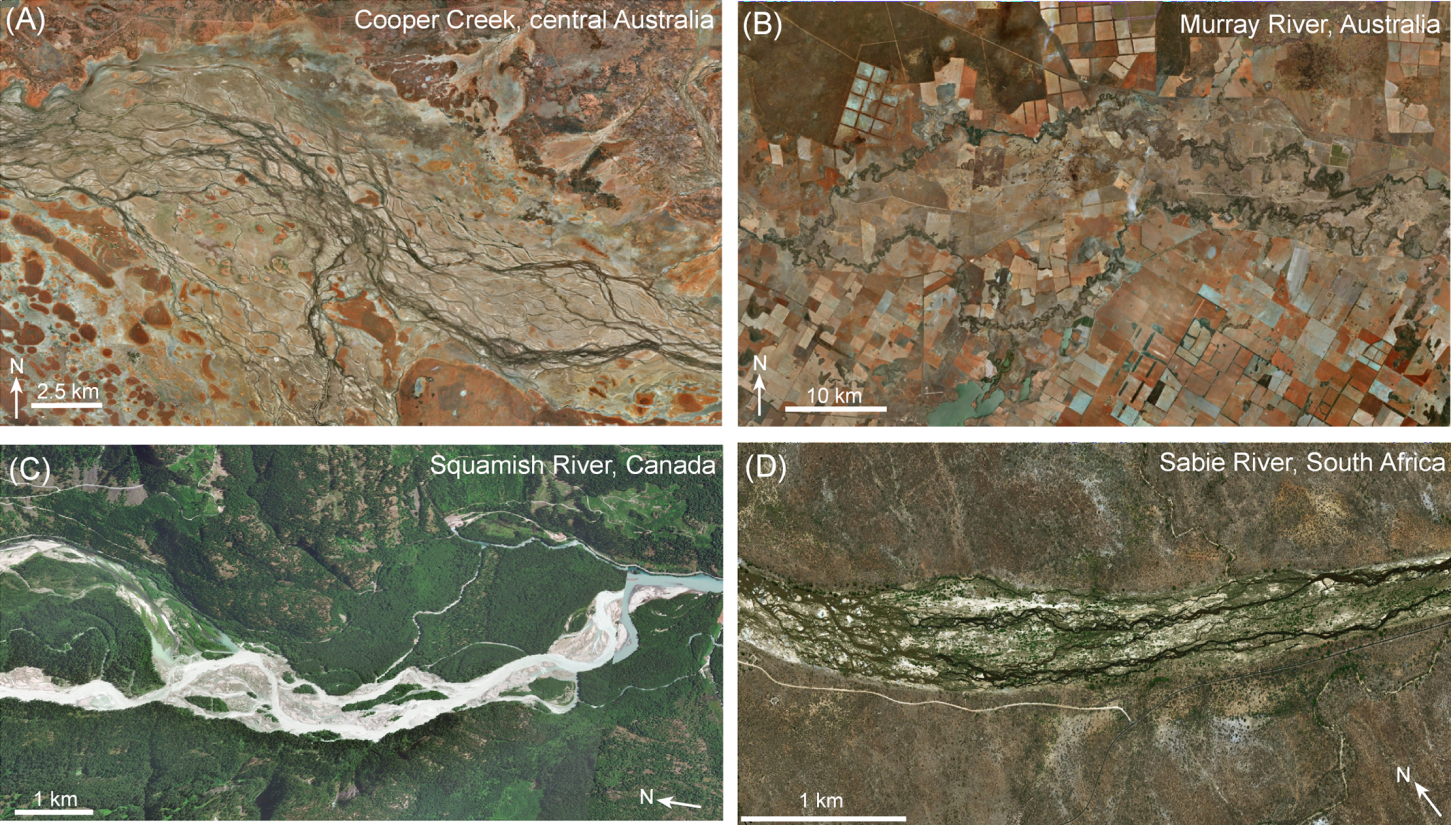
Are these all anabranching river types? See text for explanation. Basemaps produced with ArcGIS software by Esri. Sources: Esri, DigitalGlobe, GeoEye, Earthstar Geographics, CNES/Airbus DS, USDA, USGS, AeroGRID, IGN, and the GIS User Community.

**Fig 3** demonstrates some of the issues faced in differentiating and naming multi-channelled rivers (see [45, 46]). In these instances it is important to distinguish among distinctive process roles for anastomosing rivers (**Fig 3A**) relative to anabranching (**Fig 3B**) or wandering rivers (**Fig 3C**) [47, 48, 49]. Beyond this, how do we differentiate these alluvial, multi-channelled rivers from bedrock-based anastomosing rivers [50, 51] (**Fig 3D**), ensuring that we give all of them appropriate names that clearly communicate their differences?

For these various ‘meandering’, ‘braided’ and ‘anabranching’ river examples, unless we define the valley context (confined versus partly confined versus laterally unconfined valley setting), and unless we interpret geomorphic adjustments of the channel in relation to the assemblages of geomorphic units on the valley bottom, our interpretations of how and why these rivers adjust in the ways in which they do may be seriously flawed. Assigning a name is critical in communicating the distinctiveness of these river types.

For partly confined rivers that have neither fully imposed nor a fully self adjusting situation, it is important to assess the position of the channel(s) on the valley bottom, interpreting how the type and extent of confining margin impacts upon the capacity and form of river adjustment in that setting [4, 5, 38]. Some partly confined rivers are margin-controlled, meaning the potential for channel adjustment is significantly limited by bedrock or other forms of confining media (e.g. terraces, fans or anthropogenic features that impose constraints upon the channel). Other reaches are planform-controlled, meaning that the shape and position of the channel on the valley bottom (and against the valley bottom margin) is a key differentiating characteristic of the river type. Examples of these rivers are presented later in this paper.

These various examples are indicative of some of the challenges that are faced in ensuring that names used to communicate different types of river convey consistent insight into both the character and behaviour of the reach under investigation.

### The procedure: River Styles procedural tree and catchment or region specific River Styles trees

**Fig 4:**
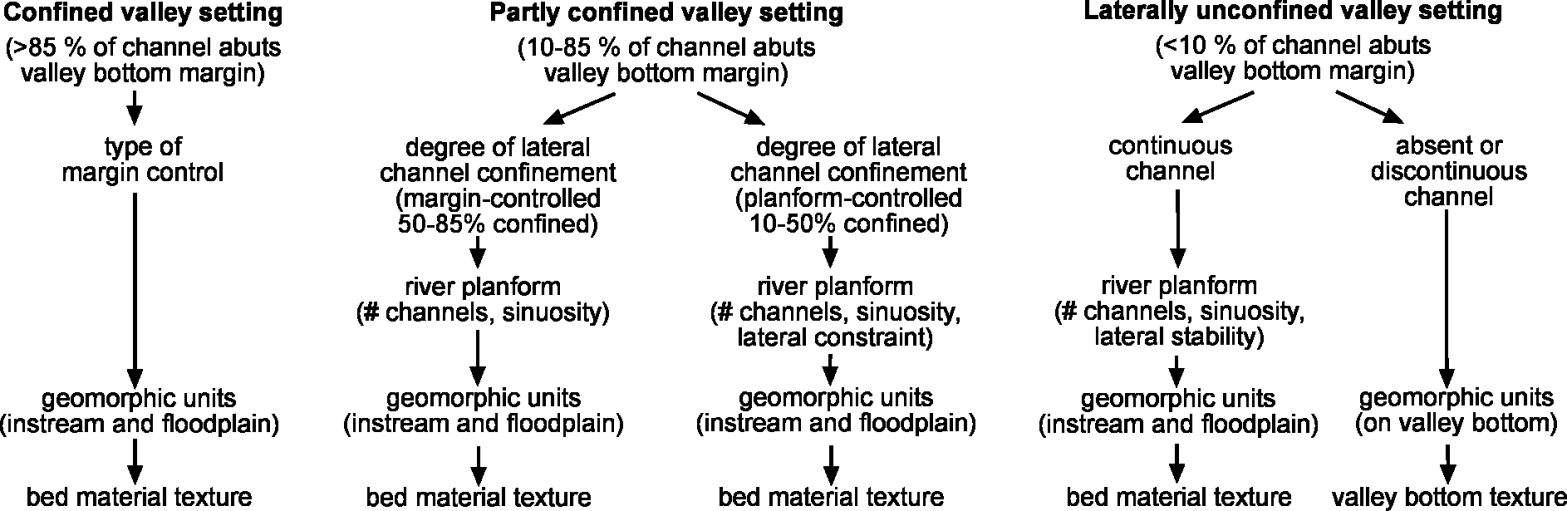
River Styles procedural tree for identifying different river types across the spectrum of river diversity. The approach identifies types of river based on a mix of measures including valley setting, channel planform, geomorphic units and bed material texture. Modified from [29] and reproduced under a CC BY license, with permission from Wiley, original copyright 2005.

The approach to identify and analyse River Styles in Stage 1 of the Framework uses three key measures; river planform, geomorphic units and bed material texture [40, 29]. These measures are ordered on the River Styles procedural tree under each valley-setting (**Fig 4**). Measures are only used in a particular valley setting if they provide useful information to help identify a river type. For example, measuring river planform in terms of the number of channels, sinuosity and form/ease of lateral channel adjustment does not provide a diagnostic indicator of river types located in confined valleys, nor for laterally unconfined discontinuous channel systems. Some measures provide important information across the spectrum of river diversity. For example, geomorphic units are the ‘building blocks’ of all rivers (e.g. [19, 29, 39, 52]). Hence, the assemblage of instream and floodplain geomorphic units is a core diagnostic indicator in the River Styles Framework. Interpretation of the assemblage of geomorphic units and their process interactions at different flow stages determines the character and behaviour of a reach.

In common with other naming procedures, the measures chosen to identify river types are structured in a hierarchical fashion. Entry into the River Styles procedural tree occurs at a relatively coarse scale through identification of valley setting. The measure used to determine valley setting is lateral confinement of the channel. This entails analysis of the position of the channel relative to a valley bottom margin, and the extent to which either channel bank (not both) abuts a valley bottom margin (see [5, 38] for automated methodology). Differentiation of floodplains, terraces, hillslopes, fans etc. follows standard geomorphic mapping techniques that identify breaks in slope between landforms and account for the shape of those landforms (e.g. see [29, 5]).

Three valley settings cover the full spectrum of river diversity; confined, partly confined and laterally unconfined. Over time, with semi-automation and testing (see [38]), the confinement categories that define each valley setting have been validated and become better defined. In the River Styles Framework, confined rivers have a channel that abuts the valley bottom margin along either bank along >85% of its length (previously >90%) (**Fig 4**). Partly confined rivers have a channel that abuts the valley bottom margin along either bank along 10–85% of its length (previously 10–90%). Finally, laterally unconfined rivers have a channel that abuts the valley bottom margin along either bank along <10% of its length. We use the term laterally-unconfined so that rivers that sit on bedrock and are not truly ‘alluvial’, but have capacity to adjust in the lateral dimension can also be identified and named. Also, some rivers in laterally unconfined valley settings have no channel or a discontinuous channel. Procedures used to differentiate among types of river are tailored to the valley setting in which they are found (see [29]). For example, in a confined valley setting the next measure used is the type of margin control, in a partly confined valley setting the next measure used is the degree of lateral channel confinement, whereas in a laterally unconfined valley setting the next measure used is the continuity of the channel. For partly confined and laterally unconfined rivers with continuous channels, river planform is then measured.

The next step in the procedural tree entails identification of geomorphic units. This is the key diagnostic indicator used for differentiating River Styles [29]. Instream and floodplain geomorphic units are pieced together (like a jigsaw) to describe the assemblage of geomorphic units that characterises that river type [53, 52]). Finally, analysis of bed material texture is used to further differentiate river types at the finest scale of resolution in the hierarchy.

The hierarchical structure of the River Styles procedural tree allows a user to exit at any stage of the process depending on the level of detail that is required for a given analysis (e.g. regional-scale analyses of river patterns, or fine-resolution habitat-scale analyses). Importantly, procedures make a sensible and useful interpretation of the type of river under investigation across these scales. Moving further through the River Styles procedural tree entails further splitting and detailed analysis, incorporating a higher degree of interpretation. Once the River Styles procedural tree has been entered, branches can occur at any level of the hierarchy/procedure. This flexibility of ‘reach identity’ bifurcations permits identification (and analysis) of the key defining characteristics of any given river type.

The River Styles procedural tree provides the structure and template atop which River Styles trees are constructed for each catchment or region under investigation [29]. Catchment or region-specific River Styles trees contain the results derived from using each measure. Details are added to each ‘box’ at each level of the tree. If the tree is exited at a certain level, the branch will end and subsequent boxes will remain empty.

As the River Styles procedure is open-ended, new variants of river type can be added, and an appropriate name assigned as required–see below. New River Styles are only added if they display characteristics that are different to what has been identified and named elsewhere. Generation of regional (or even global) trees provides a baseline to determine if a river type has previously been identified. In addition, a consistent approach is available to assess whether a new and possibly unique river type has been identified, such that procedures are available to name this type and place it in context of all other river types.

As River Styles trees visually capture a summary of the diversity of rivers in a given region, careful documentation of their characteristics enables others to use the same river type elsewhere (or add a new style if required). Ultimately, this supports direct comparisons across different regions using a consistent framework and set of data.

Importantly, designation of River Styles names using procedures outlined here does not contain descriptors of position (e.g. headwaters, lowland plain etc.), nor descriptors of landscape units (e.g. plateau, rounded foothills, delta, etc.), nor place names (see [29]). These positional or geographical attributes are not considered diagnostic indicators of river type, as some types of river can occur in different landscape positions or settings. Forcing river types into a particular landscape setting (and naming them as such) restricts generic application of the approach to naming conventions for rivers in different places. Instead, we use analyses of landscape position and setting as key measures to assess controls on downstream patterns of river character and behaviour along longitudinal profiles, across catchments and across regions (Stage 1, Step 3 of the River Styles Framework; see [29]), and for assessing (dis)connectivity impacts on river recovery potential (Stage 3 of the River Styles Framework; see [29]).

### The convention: River Styles naming convention

A well-structured, hierarchical procedural tree to identify River Styles supports direct application of a naming convention to produce consistent results and names that can be effectively transferred between places and communicated to a range of audiences. Of utmost importance is the mix and sequence of terms used to build and create a ‘reach identity’.

**Fig 5:**
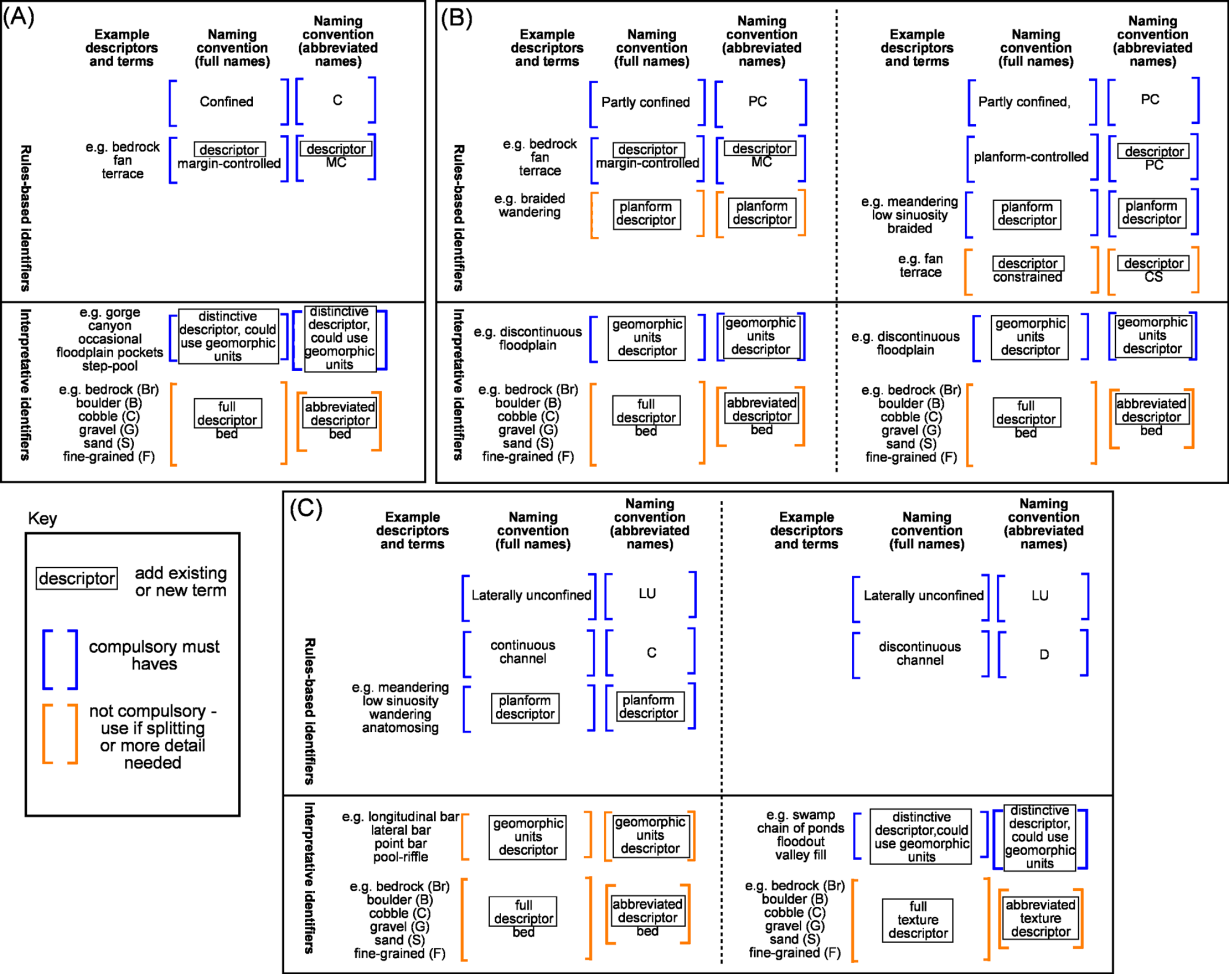
Naming convention for river types. The approach is matched to the River Styles procedural tree (Fig 4) and produces full and abbreviated names. (A) for confined valley setting, (B) for partly confined valley setting, (C) for laterally unconfined valley setting.

The naming convention for River Styles is directly linked to the River Styles procedural tree (**Figs 4 and 5**). The intent of the naming convention is to ensure that the name that is added to the base of each limb of the River Styles tree follows a consistent approach and produces a consistent name when applied in different places. In this way, users are assigning the same name to the same river type in different places rather than designing new names that prevent or inhibit direct comparisons between users or places.

The convention presented here, and the names produced, capture and communicate the key characteristics of each branch of the River Styles tree. In places where boxes such as 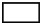 appear in the naming convention, the user is required to insert a descriptor. Where possible, existing geomorphological terms are adopted that have a ‘true’ meaning (e.g. braided, meandering), but in some cases new terms are required to adequately capture what is seen on the ground and provide a sense of how that river is behaving (e.g. planform-controlled; see [29, 4, 53]). The naming convention (**Fig 5**) provides some examples of the terms currently used for River Styles names. This is by no means all-inclusive. The words that do not appear in boxes in the naming convention are compulsory words that should be used in all River Styles names, for example the word ‘bed’ comes after differentiation of the dominant bed material texture (**Fig 5**).

The further through the River Styles procedural tree a user gets, the more interpretative the process becomes (hence the solid line across **Fig 5**). At higher levels in the hierarchy, it is now possible to automate the identification of valley-setting and river planform using rule-based identifiers (e.g. [38, 54]). At lower levels, geomorphic unit identification and bed material texture entail more interpretation and in many cases fieldwork (although approaches are being developed to semi-automate those as well; e.g. [55, 19, 56]). This rule-based versus interpretative approach to identification is incorporated in the naming convention outlined here. Hard, more consistent terms are used at higher levels, while more flexible and variable terms are communicated at lower levels (e.g. [57]).

**Fig 6:**
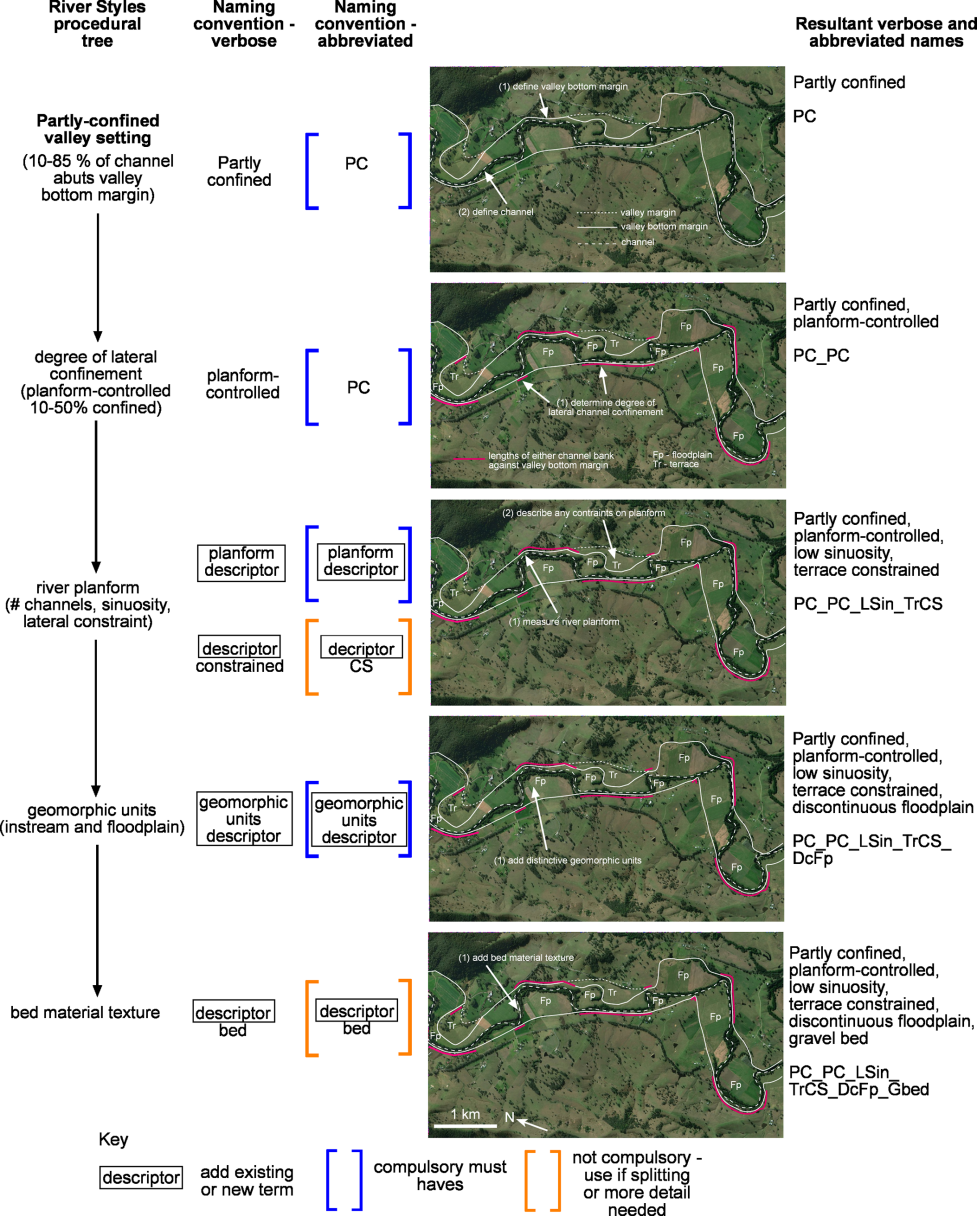
Worked example of how to use the naming convention for a partly confined river. This example demonstrates how the naming convention is hierarchical and how the approach is used to identify and name a river type. The further through the approach a user gets, the more detail is added to the identification and the name. Example is Williams River, NSW, Australia. Basemaps produced with ArcGIS software by Esri. Sources: Esri, DigitalGlobe, GeoEye, Earthstar Geographics, CNES/Airbus DS, USDA, USGS, AeroGRID, IGN, and the GIS User Community.

Just as the production of a River Styles tree for any given catchment or region can be cut off at a particular stage in the hierarchy, reflecting the specific purpose of the application, the naming of a reach can also exit or end at any stage of the sequence (**Figs 5** and **6**). A short River Styles name that only contains terms to describe valley setting and river planform, for example, will have a short name, whereas for a full analysis that includes bed material texture, the name will be longer. Hence, the length of the name can provide a direct measure of the level of detail undertaken in the analysis. Splitting or clumping can occur at any level. The further through the process a user has gone, the greater the detail needed in the name to capture the differences between river types, and therefore the longer the name. The length of the name also reflects the degree of interpretation required to arrive at that name (**Fig 6**). For example, partly confined means that a river channel is laterally confined along a significant proportion of its length and floodplain pockets occur. A partly confined, planform controlled, low sinuosity river has irregularly shaped floodplains, the morphology of which is controlled by the low sinuosity planform of the channel and its position relative to the valley bottom margins. A partly confined, planform controlled, low sinuosity, fine grained bed versus gravel bed means that of these low sinuosity variants, the fine grained type is likely to have less capacity to adjust and may contain no evidence of channel shift, whereas the gravel variant (**Fig 6**) may have capacity to adjust through downstream translation of meander bends or chute cutoffs where the channel is not abutting a valley bottom margin or terraces.

**Fig 7:**
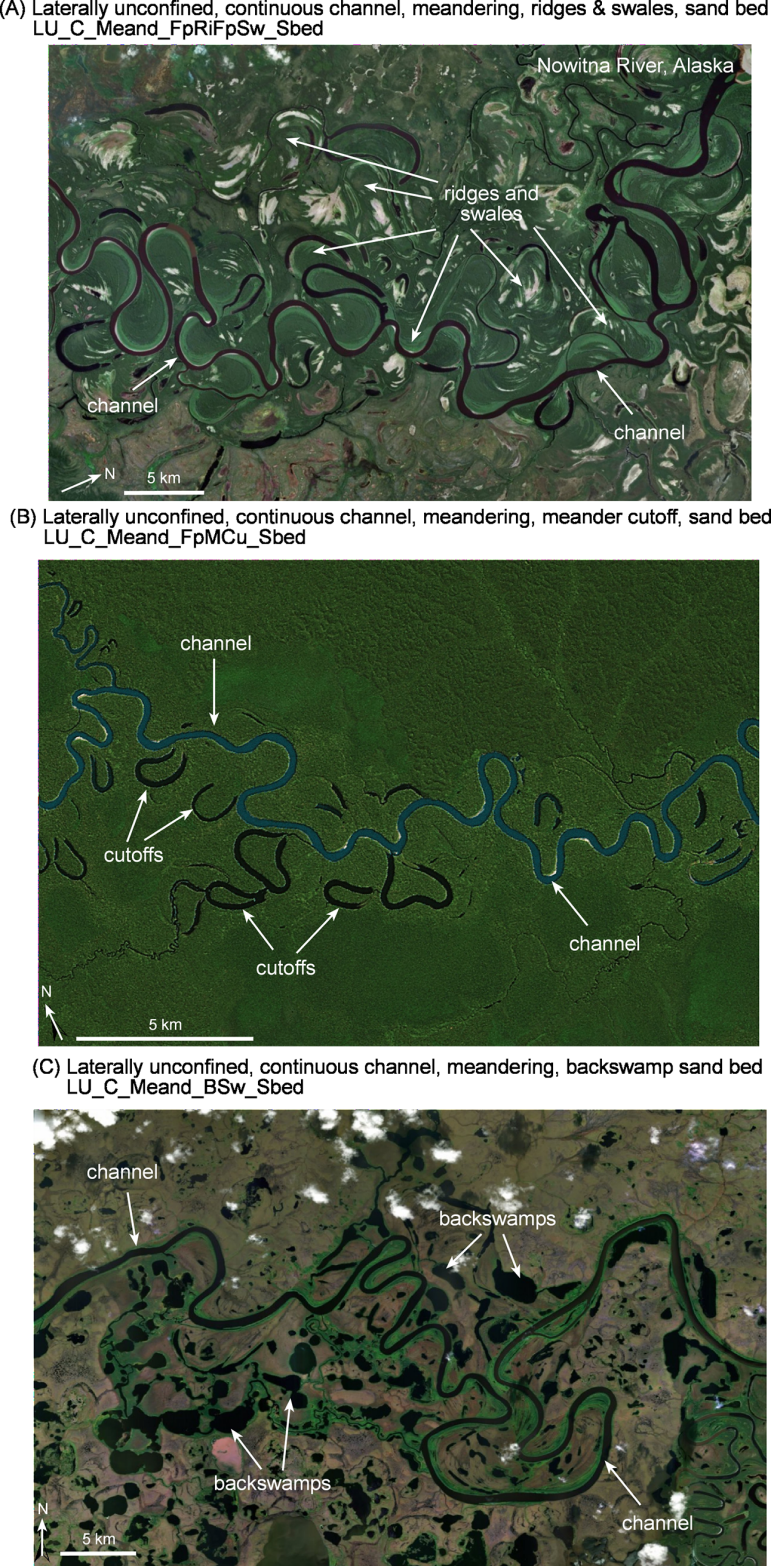
Examples of Laterally unconfined, continuous channel, meandering, sand bed rivers where using a geomorphic unit descriptor is useful for further differentiating between variants of this river type.

Irrespective of the level of detail of analysis, there are some ‘must haves’ in the naming of River Styles (**Fig 5**). These must haves relate to measures used at the entry point of the River Styles tree, as well as some specific terminology. Minimum must haves are a valley setting (C, PC or LU for confined, partly confined and laterally unconfined respectively), some descriptors of lateral confinement (MC or PC for margin-controlled or planform-controlled respectively) and river planform, and for rivers in the partly confined and laterally unconfined valley setting the words ‘discontinuous floodplains’ (DcFp) and ‘continuous channel’ (C) or ‘discontinuous channel’ (D) respectively. Additional descriptors associated with river planform and geomorphic units are only used when trying to communicate more detail or if splitting is required. **Fig 7** contains an example where the use of a geomorphic unit descriptor is useful for differentiating between different laterally unconfined, meandering, sand bed rivers where the floodplains are dominated by ridge and swale topography indicative of lateral migration (**Fig 7A**), cutoffs formed by channel avulsion (**Fig 7B**) and backswamps indicative of low lateral migration rates and vertical accretion of floodplains (**Fig 7C**). Another example may be the differentiation between a laterally unconfined, anastomosing, bedrock bed river that is vertically constrained but can adjust laterally versus a laterally-unconfined, anastomosing, fine grained bed river that has capacity to adjust both vertically and laterally. The use of a geomorphic unit descriptor (e.g. bedrock core bar and sculpted longitudinal bar, respectively) may be useful in the name.

The naming convention for rivers found in the confined valley setting has three levels, linked directly to the River Styles procedural tree (**Figs 4** and **5**). The type of margin control is assessed first. A descriptor of that margin-control becomes a compulsory ‘must have’ as part of the name (e.g. bedrock, terrace, fan etc.). At the next level geomorphic units are identified and a distinctive descriptor is added to the name. As a general rule, the dominant or key defining geomorphic unit can be used in the name (e.g. occasional floodplain pockets) as a ‘must have’. If further detail is required, then bed material texture is assessed and this is added to the name using one of six descriptors (bedrock (Br), boulder (B), cobble (C), gravel (G), sand (S), fine-grained (F)) (**Table 1**). In most cases only the dominant bed material texture is used in the name.

**Table 1:** Some abbreviations for describing types of margn control, river planform, geomorphic units and bed material texture as part of the naming convention. Note: list of geomorphic units taken from [53], [29] and [19].

TYPES OF MARGIN CONTROL OR CONSTRAINTS		PLANFORM TYPES	
Bedrock	Br	Headwater	Hw
Terrace	Tr	Gorge	Gge
Fan	Fn	Canyon	Cyn
Dune	Dn	Braided	Braid
**TYPES OF ANTHROPOGENIC MARGIN**		Meandering	Meand
Stopbank	SBk	Wandering	Wan
Constructed levee	CoLv	Low sinuosity	LSin
Embankment	EBk	Anastomosing	Anast
Bank revetment	BkRe	Anabranching	Anbr
Railroad	RaRd	Chain of ponds	CoP
Road	Rd	Valley fill	VFi
Pipe	Pip	Swamp	Swp
Concrete	Crt	Floodout	Fout
Earth	Ea	Canal	Cnl
I**NSTREAM GEOMORPHIC UNITS**			
**Sculpted, erosional geomorphic units**		**Mid-channel geomorphic units**	
Bedrock step (waterfall)	BrSt	Alluvial riffle	ARi
Step-pool	SPo	Alluvial pool	APo
Cascade	Cc	Longitudinal bar (medial bar)	LoBa
Rapid	Rp	Transverse bar (linguoid bar)	TBa
Run (glide, plane-bed)	Ru	Diagonal bar (diamond bar)	DBa
Forced riffle	FoRi	Expansion bar	EBa
Forced pool	FoPo	Island	Isl
Plunge pool	PPo	Boulder mound	BMd
Pot hole	PHo	Bedrock core bar	BrCBa
**Bank-attached geomorphic units**		Sand sheet	SSh
Lateral bar (alternate or side bar)	LaBa	Gravel sheet	GSh
Scroll bar	ScBa	Forced mid-channel bar (pendant bar, wake bar, lee bar)	FMcBa
Point bar	PtBa	Compound mid-channel bar	CMcBa
Tributary confluence bar (channel junction bar, eddy bar)	TCBa	Alluvial riffle	ARi
Ridge	Ri	Alluvial pool	APo
Chute channel	CCh	Longitudinal bar (medial bar)	LoBa
Ramp (chute channel fill)	Rp	**Fine-grained sculpted geomorphic units**	
Bench	Be	Sculpted lateral bar	SLaBa
Point bench	PtBe	Sculpted longitudinal bar	SLoBa
Ledge	Le	Sculpted point bar	SPtBa
Point ledge	PtLe	Sculpted run	SRu
Boulder berm	BBrm	Sculpted pool	SPo
Concave bank bench	CCBe		
Compound bank-attached bar	CBABa		
Forced bank-attached bar	FBABa		
**FLOODPLAIN GEOMORPHIC UNITS**			
Occasional floodplain	OccFp	Palaeochannel (prior channel, abandoned, ancestral channel)	FpPc
Discontinuous floodplain	DcFp	Ridge	FpRi
Floodplain (alluvial flat)	Fp	Swale	FpSw
Levee	Lv	Valley fill (swamp, swampy meadow)	Vfi
Crevasse splay	CSp	Meander cutoff (neck cutoff, billabong)	FpMCu
Floodchannel (back channel)	FCh	Ox bow	FpOx
Flood runner	FRu	Chute cut-off	FpChCu
Backswamp (distal floodplain, floodplain wetland, floodplain lake)	BSw	Floodplain channel anabranch (secondary or flood channel)	FpCab
Floodplain sand sheet	FpSs		
**BED MATERIAL TEXTURE**			
Bedrock	Br	Gravel	G
Boulder	B	Sand	S
Cobble	C	Fine grained	F

The naming convention for rivers in the partly confined valley setting has four levels, linked directly to the River Styles procedural tree (**Figs 4** and **5**). The first split occurs between margin-controlled rivers where either bank of the channel abuts the valley bottom margin along 50–85% of its length, and planform-controlled rivers where either bank of the channel abuts the valley bottom margin along 10–50% of its length. If the river is margin-controlled, a descriptor of the type of confining media is added to the name at the next level as a ‘must have’ (e.g. bedrock, terrace, fan, etc.). River planform is measured at the next level, assessing the number of channels and sinuosity. For the margin-controlled rivers, planform is not free-forming and is not considered a diagnostic characteristic of these river types, and therefore not compulsory in the name. However, if a descriptor is needed to add value or is needed to split between types, then it can be added to the name. If the river is planform-controlled a descriptor of planform is a compulsory ‘must have’ in the name, as the type of planform is a defining characteristic of planform-controlled rivers. If additional constraint is being applied to the river planform via secondary confining features, then these can be added to the name of planform-controlled rivers, but are not compulsory. For all partly-confined rivers, the next level of analysis is at the geomorphic unit scale. A distinctive descriptor is added to the name. The dominant or key defining geomorphic unit can be used (e.g. discontinuous floodplain). If further detail is required, then bed material texture is assessed and this is added to the name.

The naming convention for rivers found in the laterally unconfined valley setting has four or three levels depending on whether the channel is continuous or discontinuous respectively (**Figs 4** and **5**). For laterally unconfined rivers with continuous channels, river planform is a diagnostic indicator of river type. As such, it is a compulsory ‘must have’ descriptor in the name. Geomorphic units and bed material texture may be needed in the name to differentiate between different types of rivers (e.g. see **Fig 7**). For laterally unconfined rivers with discontinuous or absent channels, a distinctive descriptor is used at the geomorphic unit scale and is a compulsory ‘must have’. For these rivers, material texture is analysed for the valley bottom (as these rivers have no channels) and the label ‘bed’ is removed from the name.

### The product: Full (verbose) and abbreviated River Styles names

The intent of River Styles names has always been to capture the dominant geomorphic process-form characteristics of a river that when communicated (and said out-loud) provide an immediate image or cognitive picture that identifies what that river looks like, and by extension how it is expected to behave. For this reason, River Styles names have not been converted to codes, as applied in some river classification schemes (e.g. [58, 13, 23]). While the names can become verbose, they are considered to provide an innate meaning to practitioners. This provides the nuance that is required to distinguish between river types, helping to communicate what is seen on-the-ground. The challenge lies in developing a scheme that can produce this response through use of consistent language and terms, while also communicating the key differences between river types.

Because the ***full names*** can be long, the River Styles names can be abbreviated while still maintaining their hierarchical structure, sequencing of terms and interpretative power. The ***abbreviated names*** must always be directly linked to the full name, retaining the ability to translate one into the other. Therefore, they must follow the same convention and sequencing. In this way, the abbreviations could be considered as aliases. However, an alias can only be used after the full name has been presented and understood (https://en.oxforddictionaries.com). In some cases, the abbreviated name could appear on the River Styles tree or legends on maps, while the full names are used in River Styles proformas. Wherever abbreviations are used, a direct link should be made to the full name somewhere in a table or sub-section of a report/paper. Examples of some of the abbreviations used to describe types of margin control, planform, instream and floodplain geomorphic units and bed material texture are in **Table 1**. This table is by no means all-inclusive, and is presented here as a guide only. It is not intended to be a full set of options that are plugged in to the name, but provides a starting point for developing consistent abbreviations for some of the more common attributes of rivers used in the naming convention presented here.

The production of a ***full name*** follows the naming convention scheme with each term or descriptor separated by a comma (,) (**Fig 5**). The sequence must be consistent and include the ‘must haves’ as a bare minimum. The production of an ***abbreviated name*** follows the same convention as the production of full names and can only occur after a full name has been produced. Abbreviations of each level in the convention are separated by an underscore (_) (**Fig 5**). Again, the sequence must be consistent and include the ‘must haves’ as a bare minimum.

**Fig 8:**
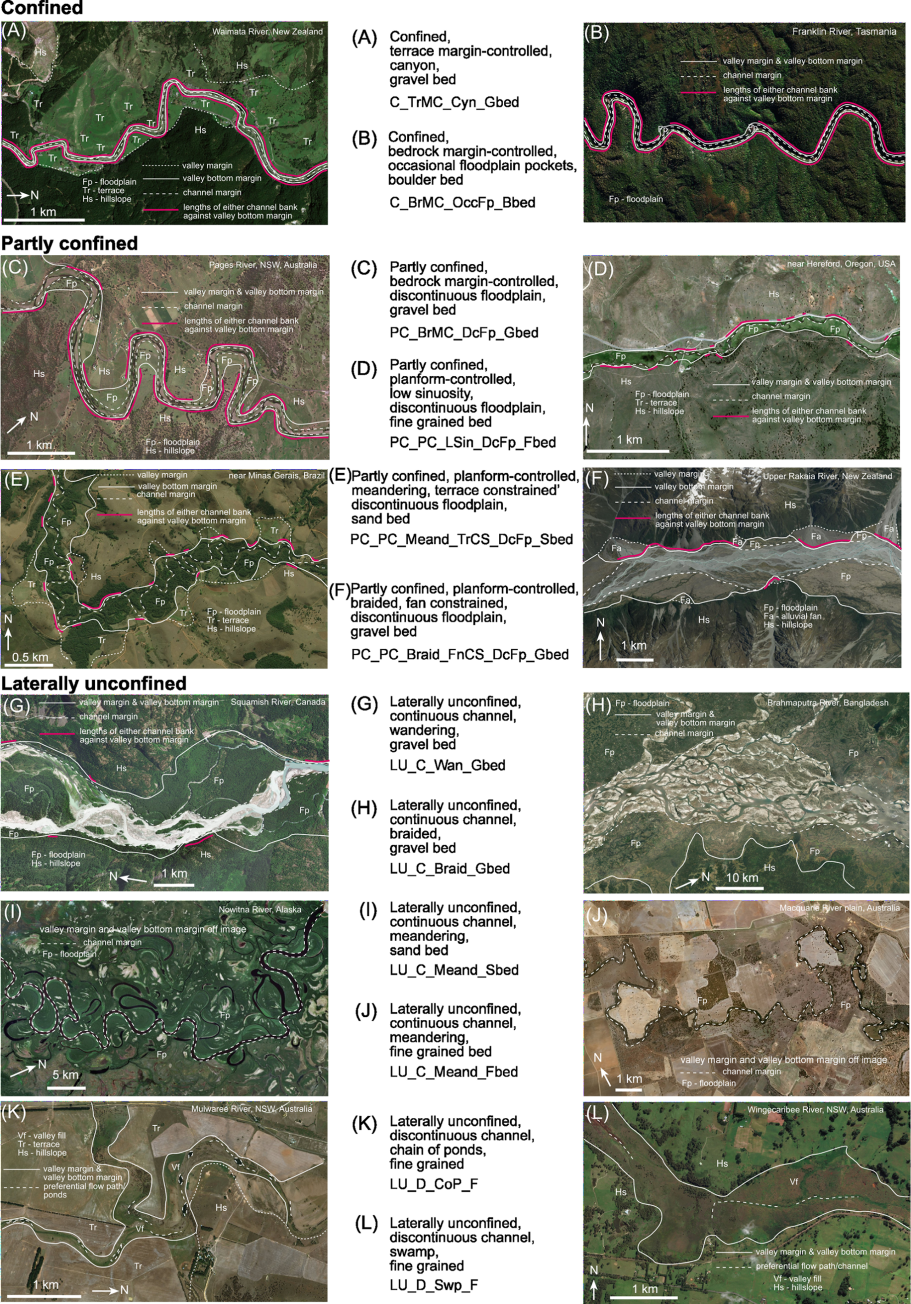
Worked examples of full and abbreviated names for a range of river types across the spectrum of river diversity. Examples are shown for confined, partly confined and laterally unconfined river types. Basemaps produced with ArcGIS software by Esri. Sources: Esri, DigitalGlobe, GeoEye, Earthstar Geographics, CNES/Airbus DS, USDA, USGS, AeroGRID, IGN, and the GIS User Community.

**Fig 8** provides examples that apply the naming convention across a range of rivers in different valley settings. Both full and abbreviated names are provided. In the confined valley setting, two rivers that look similar in terms of planform are presented. Previously these rivers may have been labelled as gorges or canyons and clumped as the same. However, by applying the River Styles procedure and assigning an adequate name (both full and abbreviated), the differences between the two become quite clear. The use of additional descriptors helps to make some initial interpretations about the character and behaviour of these two variants. **Fig 8A** is a Confined, terrace margin-controlled, canyon, gravel bed river (abbreviated to C_TrMC_Cyn_Gbed). This is quite a different river to **Fig 8B**, which is a Confined, bedrock margin-controlled, occasional floodplain pockets, boulder bed river (abbreviated to C_BrMC_OccFp_Bbed).

In the partly confined valley setting, **Fig 8C** may have previously been erroneously considered a meandering river. However, it is a margin-controlled river with little capacity to adjust. This is reflected in the name, Partly confined, bedrock margin-controlled, discontinuous floodplain, gravel bed river (PC_BrMC_DcFp_Gbed). **Fig 8D** and **8E** are variants of planform controlled rivers, one is low sinuosity and one is meandering. In **Fig 8E**, the presence and position of terraces constrains planform adjustments. The names of these rivers communicate these differences; Partly confined, planform-controlled, low sinuosity, discontinuous floodplain, fine grained bed river (PC_PC_LSin_DcFp_Fbed) and Partly confined, planform-controlled, meandering, terrace constrained, discontinuous floodplain, sand bed river (PC_PC_Meand_TrCS_DcFp_Sbed). **Fig 8F** is a river that traditionally may have been considered an alluvial braided river. Here is it considered to be a partly confined, planform-controlled river with significant constraint imposed by alluvial fans and bedrock hillslopes. This river is named Partly confined, planform-controlled, braided, fan constrained, discontinuous floodplain, gravel bed river (PC_PC_Braid_FnCS_DcFp_Gbed).

The range of river diversity in the laterally unconfined valley setting is vast. Some of the more traditional river types maintain more traditional names such as Laterally unconfined, continuous channel, wandering, gravel bed river (LU_C_Wan_Gbed) in **Fig 8G**, Laterally unconfined, continuous channel, braided, gravel bed river (LU_C_Braid_Gbed) in **Fig 8H**, and Laterally unconfined, continuous channel, meandering, sand bed river (LU_C_Meand_Sbed) in **Fig 8I**. However, by adding nuance through the bed material texture descriptor, the name can also provide significant insight into the expected geomorphic structure and behaviour of the river, as noted by the differences between the meandering sand bed river (**Fig 8I**) and the Laterally unconfined, continuous channel, meandering, fine grained bed river (LU_C_Meand_Fbed) in **Fig 8J**.

For laterally unconfined rivers that have absent or discontinuous channels, the names can be used to communicate diversity of river types that are poorly recognised. Examples shown in **Fig 8** are Laterally unconfined, discontinuous channel, chain of ponds, fine grained river (LU_D_CoP_F) in **Fig 8K** or Laterally unconfined, discontinuous channel, swamp, fine grained river (LU_D_Swp_F) in **Fig 8L**.

**Table 2** uses the naming convention procedure outlined in this paper to derive the names of the examples shown in **Figs 1–3**. While names may appear cumbersome, they capture significant differences in the character and behaviour of these examples, aiding clarity in communicating differences between these rivers.

**Table 2:** Full and abbreviated names for rivers in Figs 1–3. For examples of abbreviations to use in names, see Table 1.

Figure	Full (verbose) name	Abbreviated name
**1A**	Confined, bedrock margin-controlled, canyon, gravel bed	C_BrMC_Cyn_Gbed
**1B**	Partly confined, bedrock margin controlled, discontinuous floodplain, gravel bed	PC_BrMC_DcFp_Gbed
**1C**	Laterally unconfined, continuous channel, meandering, sand bed	LU_C_Meand_Sbed
**1D**	Laterally unconfined, continuous channel, meandering, fine grained bed	LU_C_Meand_Fbed
**2A**	Partly confined, planform-controlled, braided, terrace constrained, discontinuous floodplain, gravel bed	PC_PC_Braid_TrCS_DcFp_Gbed
**2B**	Laterally unconfined, continuous channel, braided, gravel bed	LU_C_Braid_Gbed
**3A**	Laterally unconfined, continuous channel, anastomosing, fine grained bed	LU_C_Anast_Fbed
**3B**	Laterally unconfined, continuous channel, anabranching, fine grained bed	LU_C_Anbr_Fbed
**3C**	Laterally unconfined, continuous channel, wandering, gravel bed	LU_C_Wan_Gbed
**3D**	Partly-confined, bedrock margin controlled, anastomosing, bedrock bed	PC_BrMC_Anast_Brbed

**Fig 9:**
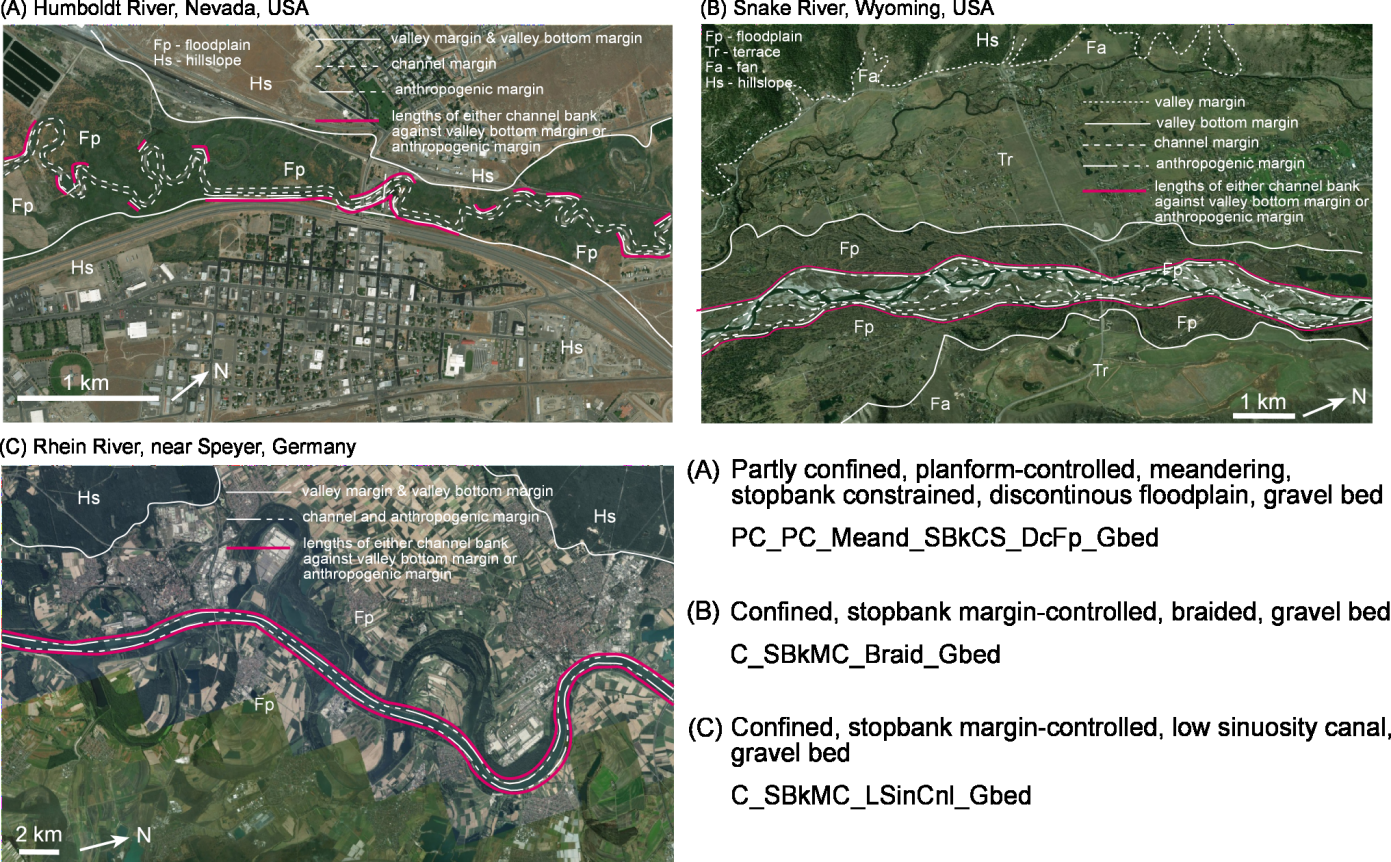
Demonstration of how the naming convention can be used for identifying and naming anthropogenically modified rivers. Basemaps produced with ArcGIS software by Esri. Sources: Esri, DigitalGlobe, GeoEye, Earthstar Geographics, CNES/Airbus DS, USDA, USGS, AeroGRID, IGN, and the GIS User Community.

The procedures outlined here can also support more consistent and insightful analyses of anthropogenically modified and urban streams. For these rivers, an added layer of confining margin is often prevalent. When identifying and naming these rivers, these anthropogenic margins (see [5]) and features can be included at two levels. First, they can be applied at the valley setting level to differentiate the type and extent of confining or constraining media. Second, they can be used at the geomorphic unit level to characterise how constructed or confining media impact upon ‘forced’ geomorphic units (or structural elements; [19]), such as stopbanks, constructed levees, railroads, roads, pipes, etc. The extent to which these features influence the character and behaviour of the river can be reflected in the name at these levels. **Fig 9** demonstrates how the naming convention procedure outlined in this paper can be extended to identify and name three variants of anthropogenically modified rivers.

## Discussion and conclusions

The naming convention for river types outlined in this paper seeks to establish a clear, articulate and precise set of procedures with which to identify and name river reaches across the spectrum of diversity. We contend that this approach to analysis and communication may aid transferability of understandings of geomorphic insights into river systems across multiple scales (i.e. within- and between-catchments at regional, national and intercontinental scales).

Any scientific naming convention builds upon core principles. First, it builds upon a sensible and tested line of reasoning/interpretation that can be used in a reliable manner, whereby a given name conveys consistent meaning to a range of practitioners. Exposure to such terminology and familiarity with it, particularly in river management, is critical for communication across audiences and users (e.g. students, public citizens, scientists, practitioners, policy-makers etc.). Second, a naming convention should capture the spectrum of diversity so that it can be used wherever a ‘type’ of entity occurs. Third, diagnostic attributes should differentiate between ‘types’. Fourth, a hierarchical structure allows applications to start at a coarse resolution scale with relatively few categories, and end at a fine resolution scale that specifies the many individual types (with prospect to add further types, as required). In procedures outlined here, splitting can occur at any level of the process. This allows a user to determine where distinctions occur (at the splits) and where common characteristics occur. Clumping can occur if the intent of the analysis only requires data on coarser groups of entities. Fifth, adjectives used within the name should have consistent meaning so the user and the audience have a common and shared language to work with [6].

This paper designs a naming convention and set of products that formalises the delivery, display and communication of types of river using the River Styles Framework [29, 53, 23]. Such formalisation provides the scaffold and place holders for differentiating and naming types of river. This facilitates the integration of field interpretations (reading the landscape; [24, 53]) with remotely-sensed analyses. These procedures can support the development and use of semi- or fully-automated approaches to analysis of riverscapes, providing logical and sequential rules to define and identify geomorphic river reaches through a combined analysis of confinement, river planform, geomorphic units and bed material texture (e.g. [59, 19, 5, 38]). Prospectively, syntheses of these applications could become embedded in the interactive construction, presentation and use of River Styles trees. The River Styles trees are communication and decision tools that summarise the diversity of river types, while providing a flexible framework and set of procedures that can be adapted to help identify the full spectrum of rivers. In a sense the River Styles procedural tree is a filtering exercise with a series of diagnostic indicators and measures that can be used to differentiate (at any scale and in any fluvial landscape) geomorphic river types. The convention presented here includes both genetically diagnostic *and* morphologically descriptive terminology [7].

In contrast to procedures for naming conventions used in biology, botany and zoology, or in soil classification and identification/naming of rock types [31], we do not feel that river types can be appropriately identified and named using classification schemes and dichotomous keys based upon yes/no (present/absent) categories (see [25]). Perhaps inevitably in reach-scale determinations, considerable overlap is possible in the composition and make-up of attributes at smaller scales of analysis. Giving the river type a name rather than a code, provides a common ground for communication [60, 61, 62]). Ultimately, procedures used to name a river reach, and appraise its associated meaning, must make sense to practitioners in the field.

In setting up and using a naming convention, it is important to consider who is the holder of this information, who administers and approves ‘new discoveries’, and who audits global databases to avoid the production of ‘unstable names’ that result from erroneous identification [31]. Once principles and procedures become embedded, it may be hard to go back on them. Perhaps the emergence of biological cybertaxonomies and repositories that are held on the web and administered by certified practitioners or professional organisations could point the way for a systematic and comprehensive approach to the identification and naming of geomorphic river types (cf., [31])?

In communicating fluvial geomorphology, a name provides a user with an expectation of what that river type looks like (its character), how it behaves (i.e. form-process associations, structural and functional elements of that river type and the capacity for that river to adjust), and insight into the controls on process that help create the morphology (e.g. types of margin-control or constraint). To a geomorphologist, the name might also provide some insight (or cues) for thinking about how that river may have adjusted in the past, its sensitivity or fragility, its future evolutionary trajectory, and controls upon that type of river. Hence, “What’s in a name?” is critical for geomorphic interpretations and communication. Giving something a name may have major implications for how we relate to, and seek to manage, that thing. This is especially pertinent in the designation of rules that underpin the automation of procedures for environmental assessment, monitoring and reporting. The ways in which we structure, contextualise and operationalise these activities will influence what we see and how we interpret it. Hence, while we hope that the convention to name types of river at the reach scale outlined in this paper provides a sound basis for such practices, we encourage practitioners to recurrently question (and never forget) the foundations and principles upon which this approach to enquiry is based. Such considerations are a vital part of a learning and adaptive approach to environmental science and management.
